# Something inside my head

**DOI:** 10.1192/j.eurpsy.2022.1912

**Published:** 2022-09-01

**Authors:** T. Jiménez Aparicio, G. Medina Ojeda, C. De Andrés Lobo, C. Vallecillo Adame, J. Gonçalves Cerejeira, I. Santos Carrasco, G. Guerra Valera, A. Gonzaga Ramírez, M. Queipo De Llano De La Viuda, N. Navarro Barriga, M. Fernández Lozano, B. Rodríguez Rodríguez, M.J. Mateos Sexmero, N. De Uribe Viloria

**Affiliations:** 1 Hospital Clínico Universitario, Psiquiatría, Valladolid, Spain; 2 Sacyl, Hospital Clínico Universitario Valladolid, Psiquiatría, Valladolid, Spain; 3 Hospital Clínico Universitario de Valladolid, Psiquiatría, VALLADOLID, Spain; 4 Hospital Clínico Universitario de Valladolid, Psychiatry, Valladolid, Spain; 5 Clinical Hospital of Valladolid, Psychiatry, Valladolid, Spain; 6 Hospital Universitario Fundación de Alcorcón, Psiquiatría, Valladolid, Spain

**Keywords:** Delusional disorder, ECT, Electroconvulsive therapy

## Abstract

**Introduction:**

Electroconvulsive therapy (ECT) is a medical treatment for those patients with high suicide risk or refractory psychiatric disorders. It is currently a safe technique, and its effectiveness has been widely demonstrated.

**Objectives:**

Presentation of a clinical case about a patient with drug-resistant delusional disorder and high suicide risk, who eventually received ECT treatment.

**Methods:**

Bibliographic review including the latest articles in Pubmed about ECT procedure, effects and use.

**Results:**

We present a 45-year-old man, who visited different doctors several times by reporting he had the feeling of “having a brain tumor or a vascular disorder”, so he requested imaging tests (computed tomography and magnetic resonance). These tests were absolutely normal, but he kept thinking something was wrong, and eventually attempted suicide by hanging (his family founded him before it was too late). The patient was admitted to hospital, and started psychopharmacological treatment, with minimal response. He desperately insisted that he had “something inside his head”. At this point, it was proposed to start ECT, and the patient accepted. After 6 bilateral ECT sessions, he was visibly more relaxed and less worried, and he no longer presented autolytic ideation. He was still a little bit suspicious about the feeling of having a neurological disease. Currently, the patient runs a follow-up consultation.

**Conclusions:**

Electroconvulsive therapy is a safe and effective technique for those patients with high suicide risk. It may be useful to perform imaging tests in certain cases, for detecting intracranial pressure, acute hemorrhage, tumors… A follow-up of these patients must be performed

**Disclosure:**

No significant relationships.

